# Multicenter Diagnostic Accuracy Evaluation of the Luminex Aries Real-Time PCR Assay for Group B Streptococcus Detection in Lim Broth-Enriched Samples

**DOI:** 10.1128/JCM.01768-17

**Published:** 2018-07-26

**Authors:** D. R. Hernandez, D. M. Wolk, K. L. Walker, S. Young, R. Dunn, S. A. Dunbar, A. Rao

**Affiliations:** a Geisinger Health, Danville, Pennsylvania, USA; b Scott and White Medical Center, Baylor Scott and White Health, Temple, Texas, USA; c TriCore Reference Laboratories, Albuquerque, New Mexico, USA; d Luminex Corporation, Austin, Texas, USA; Cleveland Clinic

**Keywords:** Aries, GBS, Luminex, Streptococcus, group B Streptococcus, molecular methods

## Abstract

The vertical transmission of group B Streptococcus (GBS) strains causing neonatal sepsis is one of the leading reasons for neonatal mortality worldwide. The gold standard for GBS detection is enriched culture with or without the aid of chromogenic agars. Given the high risk for morbidity and mortality in this population, high assay sensitivity is required to prevent the personal and economic costs of GBS disease. Nucleic acid amplification tests (NAATs) allow for objective determination of GBS colonization with a sensitivity and a specificity higher than those of traditional culture methods. In this study, we determined the analytical and clinical performance of the Aries GBS assay compared to those of the enrichment culture method, biochemical identification, and the NAATs used at the study sites. Remnant Lim broth samples were used to perform the Aries assay and reference testing. Upon first testing using enriched culture as the reference standard, the Aries GBS assay identified GBS with a 96.1% sensitivity (95% confidence interval [CI], 91.2 to 98.7%) and a 91.4% specificity (95% CI, 88.8 to 93.6%). The test performed with 100% positive agreement (95% CI, 83.2 to 100%) compared to the results of the BD Max GBS assay and 98.0% positive agreement (95% CI, 89.2 to 99.9%) compared to the results of the Cepheid Xpert GBS LB test. Repeatability and reproducibility were maintained in intra- and interlaboratory testing, regardless of the instrument, module, or user who performed the test. The Aries GBS assay can be set up in less than 5 min and produces results in 2 h. The easy setup, with minimal hands-on time, and high assay sensitivity and specificity make this a useful testing option for GBS screening in prepartum women.

## Abstract (precis)

The vertical transmission of group B Streptococcus (GBS) strains causing neonatal sepsis is one of the leading reasons for neonatal mortality worldwide. The gold standard for GBS detection is enriched culture with or without the aid of chromogenic agars.

## INTRODUCTION

Despite the presence of guidelines that describe screening and prophylactic treatment practices for Streptococcus agalactiae (group B Streptococcus [GBS]) colonization in pregnant women, infection with S. agalactiae remains a leading cause of morbidity and mortality in neonates born in the United States (1, 2). GBS colonizes the vagina and/or rectum, can be transmitted to a fetus during gestation, and can cause preterm delivery, stillbirths, and puerperal sepsis (3). GBS can also be transmitted to the newborn during birth, causing either early-onset (birth to day 6) or late-onset (7 to 89 days from birth) disease, which can present as pneumonia, meningitis, or sepsis (1, 3).

Current guidelines to prevent GBS disease in neonates rely on culture-based screening of pregnant women at 35 to 37 weeks of gestation (4). Vaginorectal samples are enriched in Todd-Hewitt broth with colistin and nalidixic acid (Lim broth) or other suitable media for 18 to 24 h, followed by inoculation on sheep's blood agar (SBA), identification of GBS-like colonies, and confirmation by agglutination testing for streptococcus grouping or by the CAMP test (4, 5). The entire protocol has several limitations, including the time from enrichment to the subjective identification of colonies on SBA and the relatively low sensitivity of culture, which ranges from 54 to 90% (6–9). Identification of GBS colonies is based on the presence of gray pigmentation or beta-hemolysis; however, approximately 5% of all GBS strains are nonhemolytic and/or nonpigmented (NHNP) and are still capable of causing invasive disease in neonates (10). Chromogenic agars have been developed to improve the culture-based identification of GBS; however, they also have poor sensitivity, and some of them can miss NHNP GBS colonies (11). Limitations in broth-enriched culture methods can lead to the undercalling of GBS colonization.

Nucleic acid amplification tests (NAATs) for GBS detection with a sensitivity and a specificity comparable to or better than those of the gold standard, which makes them an attractive option for generalized screening, have been developed (2). To date, no direct-from-sample test has been able to match the performance of testing by either culture or a NAAT after broth enrichment; therefore, direct testing has not been widely adopted (2, 12, 13). It is estimated that using NAATs after enrichment results in 218 fewer cases of early-onset GBS disease per million births, as well as 6 fewer deaths or cases of disability in infants per million births (14).

The Aries GBS assay targets a genomic region downstream from the S. agalactiae *cfb* gene and uses the Luminex Corporation's MultiCode real-time PCR chemistry in combination with Aries systems. The Aries system performs automated nucleic acid extraction and purification, PCR, and real-time detection of the target nucleic acid sequences. The Aries GBS assay includes a sample processing control (SPC) that is automatically added to the sample chamber of the cassette to control for sample lysis, recovery of extracted nucleic acid, detection of inhibitory substances, and confirmation of PCR reagent integrity. In this study, the performance of the Aries GBS assay for the detection of GBS in Lim broth-enriched vaginorectal swab samples from antepartum pregnant women was compared to that of the culture and agglutination-based procedure and, where applicable, other PCR methods.

## MATERIALS AND METHODS

### Site selection.

Prospective clinical specimens were collected and tested at three clinical sites located in the United States: (i) TriCore Reference Laboratories in Albuquerque, NM, a regional reference laboratory providing services to 14 hospitals; (ii) the Scott and White Medical Center in Temple, TX, a tertiary/quaternary care facility with 600-plus beds in the Baylor Scott and White Healthcare System; and (iii) the Geisinger Medical Center in Danville, PA, a 597-bed quaternary care hospital that is part of an integrated delivery network of 8 hospitals. These sites were geographically distinct and represented testing environments in which the device could reasonably be used by the laboratory scientists who will perform the test in clinical practice. To include the residual deidentified patient specimens in the study, an exemption from the requirement for informed consent was granted by each site's institutional review board (IRB) or human research review committee (HRRC); TriCore HRRC number 13-639, Scott and White Medical Center IRB number 160114, and Geisinger Medical Center IRB number 2014-0205. Demographic information, including age, geographic location, and the number of gestation weeks, was included with the specimen.

### Analytical performance testing. (i) Aries GBS assay procedure.

After thorough vortexing of the Lim broth sample, 200 μl was dispensed in the sample chamber of the Aries GBS assay cassette. The cassette sample chamber was capped, followed by the removal of the foil seal on the sample side of the cassette. The cassette was then inserted into the Aries instrument's magazine, which has a capacity for 6 tests. The Aries system includes a barcode reader to program the sample identification and the assay type for a run. After entering the sample identifiers and specifying the program to be run, the magazine was introduced into the Aries instrument and the run was started.

### (ii) Interassay variability.

Between-laboratory reproducibility was evaluated at clinical sites using a panel prepared by spiking cultured GBS (NATrol GBS positive control; Zeptometrix NATtrol S. agalactiae) into Lim broth at four different densities: negative, high negative (0.8 time the limit of detection [LoD]), low positive (1.5 times the LoD), and high positive (100.0 times the LoD). Three replicates of each panel member and controls were prepared and distributed to the testing sites. The samples were then processed and tested with the Aries GBS assay by two independent operators who were blind to the content for five nonconsecutive days, using one instrument at each site (i.e., 2 operators × 3 replicates × 5 days × 3 sites = 90 results per concentration for each analyte). Within-laboratory, interlot (data not shown), and interinstrument reproducibility was evaluated using the same panel of contrived specimens that was used in the reproducibility study described above.

### (iii) Intra-assay variability.

The test panel described above was also used to assess intra-assay variability (within-laboratory repeatability), where each sample was tested by the sponsor in duplicate by two operators blind to the content twice daily for 12 nonconsecutive days, for a total of 96 results for each GBS concentration tested (i.e., 2 replicates × 2 operators × 2 times per day × 12 days = 96). Testing was conducted across three Aries GBS assay cassette lots and three Aries instruments.

### (iv) Specimen stability.

Specimen stability was determined by the sponsor using samples consisting of cultured GBS (serotype Ib) spiked into Lim broth at four density levels: moderate-positive, low-positive, high-negative, and negative concentrations. Fresh samples were stored at both ambient (18°C to 25°C) and refrigerated (2°C to 8°C) temperatures and tested in triplicate with the Aries GBS assay at 4, 8, 24, and 30 h. The samples stored at a refrigerated temperature were evaluated for stability by testing in triplicate at 1, 3, 7, and 15 days. Frozen specimens were stored at −65°C to −95°C and tested in triplicate at 1 day and 1, 2, 3, and 4 months.

### Clinical performance testing. (i) Specimen inclusion/exclusion criteria.

Specimens were included in the study if they were collected from females at 35 to 37 weeks of gestation, the age of the woman was available, the original sample was collected in an appropriate liquid transport medium (Stuart or Amies) and enriched in Lim broth for ≥18 h to ≤24 h, and there was sufficient volume for testing after enrichment (>1.75 ml).

### (ii) Data exclusion criteria.

Specimens with inconclusive latex agglutination results, which were weak agglutination or agglutination that occurred in more than one latex reagent per the instructions included with the latex agglutination test kit, were excluded from analysis. Samples that had an insufficient quantity of enrichment culture to complete the study requirements or that were found to not have been tested in accordance with the provided kit instructions were also excluded from the final data analysis set.

### (iii) Sample processing.

A total of 726 clinical specimens from antepartum pregnant women were collected (as part of routine care) from May to August 2016. A single swab specimen was obtained per subject, but multiple measurements were taken per enriched culture specimen (excluding allowable reruns). The vaginorectal swab specimens were enriched in Lim broth for 18 to 24 h at 35 to 37°C. After enrichment, an aliquot of the Lim broth was used for standard-of-care (SOC) testing per each institution's procedures. The residual deidentified Lim broth was used for the reference culture method and for Aries GBS assay testing. Aries GBS assay testing was performed as described above. All operators were blind to the reference/comparator method results.

### (iv) Reference method.

Reference culture testing was performed on-site at the clinical sites in accordance with published CDC guidelines, in which enriched Lim broth aliquots were subcultured to 5% SBA and incubated for 18 to 24 h at 35 to 37°C in a 5% CO_2_ incubator. The SBA plates used by all sites were BD BBL plates from Becton, Dickinson and Company (Franklin Lakes, NJ). SBA plates with no colonies observed at 24 h were incubated for an additional 24 h before being reported as negative. Suspected GBS colonies (both hemolytic and nonhemolytic) were tested with the catalase reagent, and a Gram stain was performed. Catalase-negative, Gram-positive cocci were then confirmed to be GBS by latex agglutination (PathoDx; Remel, Kent, UK).

### (v) Comparison with other NAATs.

To further assess whether the Aries GBS assay performs comparably to other NAATs, analysis of the study data was conducted using NAAT results generated at the clinical sites as part of their SOC. Two of the clinical sites used FDA-cleared NAATs as part of the SOC: a BD Max GBS assay (catalog number 441772) for site 1 and a Cepheid Xpert GBS LB assay (catalog number GXGBSLB-10) for site 2. Site 3 used a laboratory-developed test as part of its SOC and was excluded from the NAAT comparison.

### (vi) Arbitration method.

Any specimens with discordant results between the Aries GBS assay and the reference culture method were assessed by bidirectional sequencing using analytically validated primers directed against nucleotides distinct from those targeted by the Aries GBS assay. Specimens were subjected to bidirectional sequencing using M13 forward and reverse primers (proprietary sequences) and the Sanger dideoxy sequencing method to retrieve DNA sequences. Following extraction by the bioMérieux NucliSENS easyMAG extraction method, specimens were subjected to reverse transcription-PCR using a Qiagen HotStarTaq PCR kit (Qiagen, Hilden, Germany). Sample electrophoresis and sequencing analysis were performed on a 3730xl analyzer (Thermo Fisher) using 3730xl Data Collection software (v 3.1.1) and Sequencing Analysis software (v 5.4). Sequences that were at least 200 bases in length, that had a Phred score greater than or equal to 20 for at least 90% of the bases, and that contained fewer than 5% ambiguous base calls were considered for further analysis using the BLAST program (NCBI). Acceptable matches to reference sequences by analysis with the BLAST program were those with greater than 95% query coverage and identity and an expected value (E value) of less than 10 to 30 when they were compared to the reference sequence.

### Statistical analysis. (i) Sample size determination.

Approximately 10 to 30% of pregnant women are colonized with GBS in the vagina or rectum. On the basis of an estimated GBS prevalence of approximately 20% in the clinical study, it was anticipated that the inclusion of approximately 750 specimens would be sufficient to obtain at least 150 positive specimens for determination of clinical sensitivity. Thus, in order to ensure that the study was statistically powered, a sufficient number of prospectively collected specimens was included to achieve at least 95% sensitivity with a two-sided 95% confidence interval (CI) with a lower bound of >90%.

### (ii) Data analysis.

An independent data management group analyzed the study data using validated data management programs compliant with 21 CFR Part 11. SYNCT UDP desktop software (v 1.1, build 124) was used by qualified Luminex personnel to generate line data from the raw Aries GBS assay run data files, including assay calls (positive, negative, or invalid). All calculations and analyses were performed using SAS software (v 9.2 or higher) resident on the SAS server running the Windows server 2008R2 standard operating system.

Clinical performance determinations (diagnostic sensitivity and specificity) were based on the fraction of positive (or negative) results by the reference method (culture) which were also positive (or negative) by the Aries GBS assay. Sensitivity and specificity were calculated according to standard practice (15). The 95% confidence intervals were calculated by the SAS Proc Freq procedure using the F distribution method (16). Substratification and subanalyses of the results by testing site and age group were also performed. In addition, a supplemental analysis of the results by specimen state (fresh versus frozen) was performed.

## RESULTS

### Analytical performance. (i) Limit of detection and inclusivity.

A minimum of six 2-fold serial dilutions with 20 replicates each were made for each GBS strain tested, and the concentrations were verified by colony counting. Depending on the serotype and strain tested, LoDs ranged from 8.2 × 10^2^ to 1.4 × 10^4^ CFU/ml (data not shown) (17, 18). The highest determined LoD (1.4 × 10^4^ CFU/ml for serotype II strain ATCC BAA-1175) was considered the assay LoD and was used as the basis for the reproducibility studies (described below): plating and colony counting to determine the number of CFU per milliliter and the LoD at a concentration that was detected for >95% of the samples tested. Other serotypes and strains, not included in the LoD determination, were tested for inclusivity at concentrations of 2 to 3 times the assay LoD (data not shown) (17, 18).

### (ii) Analytical specificity.

Cross-reactivity was not observed for any of the 113 microorganisms tested with the Aries GBS assay (data not shown). Likewise, none of the 28 substrates tested interfered with the assay (17, 18).

### (iii) Inter- and intralaboratory reproducibility.

Results for the inter- and intralaboratory reproducibility studies are summarized in Table 1. These results met the predefined acceptance criterion (95% correct results) for the various panels tested.

**TABLE 1:** Inter- and Intralaboratory reproducibility of the Aries GBS assay^*a*^

Panel	Interlaboratory															Intralaboratory								
	Overall			Site 1				Site 2				Site 3				Overall			Operator 1			Operator 2		
	No. of specimens positive/total no. tested	% positive	Avg *C_T_*	No. of specimens positive/total no. tested	% positive	Avg *C_T_*	*C_T_* % CV	No. of specimens positive/total no. tested	% positive	Avg *C_T_*	*C_T_* % CV	No. of specimens positive/total no. tested	% positive	Avg *C_T_*	*C_T_* % CV	No. of specimens positive/total no. tested	% positive	Avg *C_T_*	No. of specimens positive/total no. tested	% positive	Avg *C_T_*	No. of specimens positive/total no. tested	% positive	Avg *C_T_*
N	0/90	0	NA	0/30	0	NA	4.2	0/30	0	NA	4.1	0/30	0	NA	4.7	0/96	0	NA	0/48	0	NA	0/48	0	NA
HN	46/90	51.1	38.5	18/30	60	38.4	1.8	11/30	36.7	38.7	1.6	17/30	56.7	38.5	1.5	75/96	78.1	38.1	37/48	77.1	38.2	38/48	79.2	38
LP	81/90	90	37.5	26/30	86.7	37.6	2.3	29/30	96.7	37.2	1.5	26/30	86.7	37.8	1.6	92/96	95.8	37.4	45/48	93.8	37.5	47/48	97.9	37.3
HP	90/90	100	29.9	30/30	100	29.8	1.3	30/30	100	29.9	1	30/30	100	29.9	0.9	96/96	100	29.9	48/48	100	30	48/48	100	29.8

a GBS strain CDC 2008232729 was used. CV, coefficient of variation; N, negative; HN, high negative; LP, low positive; HP, high positive; NA, not applicable.

### (iv) Specimen stability.

Results for specimen stability studies (Table 2) demonstrate that the storage of fresh specimens at ambient temperatures for up to 18 h does not have a major effect on Aries GBS assay performance. The data shown for GBS-negative specimens is the mean internal control (SPC) threshold cycle (*C_T_*) value. On the basis of the findings of the stability studies, Lim broth-enriched specimens can be stored at 18°C to 25°C for up to 24 h or at 2°C to 8°C for up to 7 days. The enriched specimens or any leftover enriched specimen can be stored at −70°C or colder temperatures for up to 3 months (18).

**TABLE 2:** Specimen stability results at room temperature^*b*^

Target concn	Time point (h)	% GBS positivity (no. of specimens positive/total no. tested)	Mean *C_T_* ± SD
GBS moderate positive	0	100 (3/3)	29.3 ± 0.1
	4	100 (3/3)	28.7 ± 0.1
	8	100 (3/3)	28.2 ± 0.2
	22	100 (3/3)	26.2 ± 0.1
	30	100 (3/3)	23.8 ± 0.3
	Overall	100 (15/15)	
GBS low positive	0	100 (3/3)	37.0 ± 0.7
	4	100 (3/3)	36.5 ± 0.5
	8	100 (3/3)	35.4 ± 0.2
	22	100 (3/3)	33.8 ± 0.3
	30	100 (3/3)	33.7 ± 2.3
	Overall	100 (15/15)	
GBS high negative	0	67 (2/3)	38.3 ± 0.6
	4	100 (3/3)	37.1 ± 0.2
	8	100 (3/3)	36.1 ± 0.3
	22	100 (3/3)	35.2 ± 0.5
	30	100 (3/3)	33.2 ± 0.5
	Overall	93 (14/15)	
GBS negative^*a*^	0	0 (0/3)	31.8 ± 0.1
	4	0 (0/3)	31.5 ± 0.5
	8	0 (0/3)	32.0 ± 0.3
	22	0 (0/3)	31.2 ± 0.3
	30	0 (0/3)	32.8 ± 2.9
	Overall	0 (0/15)	

a The data shown for GBS negative are the mean SPC *C_T_* values.

b The control strain was CDC 2008232729.

### Clinical performance. (i) Sample accrual and demographics.

A total of 726 specimens were collected during the time of the study. The most common reason for sample exclusion was due to gestational age outside the 35 to 37 weeks, as recommended in GBS screening guidelines (16 specimens). Inoculation of Lim broth after 4 or more days from the time of collection excluded three specimens. Five specimens were excluded due to inconclusive reference testing results (positivity for more than 1 serogroup or weak reactions on the PathoDx streptococcal grouping kit). Lastly, 14 specimens were excluded from the analysis due to deviations on reference testing (because the plates were not read in time, because of the overgrowth of unknown organisms, because of an inability to isolate possible Streptococcus spp., etc.). The final 688 clinical specimens were used for analysis of the clinical data set. The minimum age recorded was 15 years, the median and mean ages were 27 years, and the maximum age was 44 years. No statistical significant differences in the age ranges were found between the three sites conducting the study (data not shown).

### (ii) GBS culture results.

A total of 299 samples exhibited growth on the primary blood agar plate (BAP) after Lim broth enrichment. Over 80% of the plates exhibited overgrowth and required subculturing to another BAP for colony isolation and accurate identification of streptococcal colonies. The presence or absence of hemolysis was not a factor that excluded samples from further testing; GBS was isolated from 129 clinical specimens, with the isolates from 3 specimens being nonhemolytic. The streptococcal group for the remaining 188 isolated colonies was determined by latex agglutination, resulting in 129 GBS isolates, 7 group F streptococcus isolates, and 1 group G streptococcus isolate.

### (iii) Aries GBS assay testing results.

Of the 688 clinical specimens included in the clinical study analysis, 674 (98.0%) generated valid Aries GBS assay results (i.e., positive or negative) on the first attempt. There were 14 specimens (14/688; 2.0%) that were retested because they yielded invalid results in the initial run. Thirteen of the specimens in question generated valid results upon repeat testing. The result for one specimen (01027) remained invalid upon retesting; the result for this specimen was reported as invalid in the device performance tables. A total of 647 specimens were tested by the Aries GBS assay from the fresh state (2°C to 8°C) within 24 h following Lim broth enrichment completion. The remaining 41 specimens were tested by the assay from the frozen state (≤−70°C) within 7 days following Lim broth enrichment completion.

### (iv) Diagnostic accuracy.

The clinical performance of the Aries GBS assay is summarized in Table 3. Using culture as the reference standard, the overall prevalence of GBS during the study was 18.8% (95% CI, 16.0 to 21.9%). Agreement between the two methods was 92.3%, with a sensitivity and a specificity of 96.1% and 91.4%, respectively. Discordant results between the Aries GBS assay and the reference culture method were further analyzed by bidirectional sequencing targeting genomic regions distinct from those targeted by the Aries GBS assay. The results showed that 45 of 48 specimens that were positive by the Aries GBS assay but negative by the reference culture method were positive by sequence analysis and 2 of 5 specimens that were negative by the Aries GBS assay but positive by the reference culture method were negative by sequencing.

**TABLE 3:** Performance of Aries GBS assay compared to culture after LIM broth enrichment

Site	Culture result	No. of samples with the following Aries GBS assay result:			Sensitivity (%)	Specificity (%)	PPV^*c*^ (%)	NPV^*d*^ (%)	Prevalence (%)	Agreement (%)
		Positive	Negative	Total						
Overall					96.10 (91.2–98.7)^*e*^	91.40 (88.8–93.6)	72.1 (65.0–78.3)	99 (97.7–99.6)	18.8 (16.0–21.9)	92.3 (90.0–94.1)
	Positive	124	5^*b*^	129						
	Negative	48^*a*^	510	558						
	Total	172	515	687						
Site 1					100.00 (78.2–100.0)	94.70 (88.1–98.3)	75 (53.1–88.8)	100 (95.9–100.0)	13.6 (8.4–21.3)	95.5 (89.8–98.0)
	Positive	15	0	15						
	Negative	5	90	95						
	Total	20	90	110						
Site 2					91.50 (79.6–97.6)	93.00 (87.4–96.6)	81.1 (68.6–89.4)	97.1 (92.7–98.9)	24.9 (19.2–31.5)	92.6 (88.0–95.5)
	Positive	43	4	47						
	Negative	10	132	142						
	Total	53	136	189						
Site 3					98.50 (92.0–100.0)	89.70 (85.9–92.8)	66.7 (56.9–75.2)	99.7 (98.1–99.9)	17.3 (13.8–21.3)	91.2 (88.0–93.7)
	Positive	66	1	67						
	Negative	33	288	321						
	Total	99	289	388						

a Forty-five of 48 of the Aries GBS assay-positive specimens that were negative by the reference method were positive by the bidirectional sequencing analysis.

b Two of five of the Aries GBS assay-negative specimens that were positive by the reference method were negative by the bidirectional sequencing analysis.

c PPV, positive predictive value.

d NPV, negative predictive value.

e Values in parentheses are the 95% CI.

### (v) Comparison with other NAATs.

The results of the subanalysis in which the performance of the Aries GBS assay was compared to that of other NAATs (Table 4) demonstrated that the performance of the Aries GBS assay is comparable to that of two FDA-cleared NAATs, the BD Max GBS assay and the Cepheid Xpert GBS LB assay, with the rate of positive agreement between the assays analyzed being over 98% and the rate of negative agreement being over 96%. One limitation to this analysis is that enriched cultures may have been held refrigerated for up to 24 h or frozen for up to 7 days after SOC testing until Aries GBS assay testing was performed. However, the results for the SOC molecular assays and the Aries GBS assay were treated equally, and therefore, no significant bias for one method over another occurred. Additionally, these tests are not quantitative, and small variations during storage did not specifically alter any results.

**TABLE 4:** Performance of Aries GBS assay compared to other FDA-cleared NAATs

Site, assay, and assay result	No. of samples with the following Aries GBS assay result:			% of samples with:	
	Positive	Negative	Total	Positive agreement	Negative Agreement
Site 1, BD Max GBS assay				100 (83.2–100)^*b*^	100 (96.0–100)
Positive	20	0	20		
Negative	0	90	90		
Total	20	90	110^*a*^		
Site 2, Cepheid Xpert GBS LB assay				98.00 (89.2–99.9)	96.40 (91.9–98.8)
Positive	48	1	49		
Negative	5	135	140		
Total	53	136	189		

a One specimen (specimen 01027) generated an invalid result by the Aries GBS assay after an allowable rerun. This specimen was negative by the BD Max GBS assay and was excluded from the device performance calculations.

b Values in parentheses are the 95% CI.

## DISCUSSION

This study was designed to determine the analytical and clinical performance characteristics of the Aries GBS assay. The test was capable of detecting GBS in Lim broth at concentrations above 10^3^ CFU/ml, regardless of the serotype of the bacteria or their hemolytic characteristics. The LoD for the Aries GBS assay is higher than that of some other NAATs; however, due to broth enrichment, this does not impact assay performance like it does for other methods. Since the Aries GBS assay does not require a separate extraction before amplification, the tech time is reduced compared to that of other assays, but this did not have a significant effect on assay agreement with other PCR methods (19–21). The Aries GBS assay performed with a 2.0% invalid rate on the first testing attempt. Even though the invalid rate is higher than that of other comparable NAATs, like the Cepheid Xpert GBS LB or the AmpliVue GBS assays (0.9% and 0.3%, respectively), all but one specimen yielded a valid result on retesting (20, 22). The reproducibility study determined that the Aries GBS assay has excellent inter- and intralaboratory reproducibility, regardless of the instrument, module, or technician processing the samples. The Aries GBS assay also demonstrated high analytical specificity, in that there was no cross-reaction with over 100 microorganisms tested or interference by substances commonly found in GBS screening specimens (17, 18).

GBS culture of the clinical samples tested resulted in the identification of 126 isolates with a typical morphology and hemolysis. Nevertheless, we also identified three nonhemolytic GBS isolates that could have been easily missed if the technicians had focused only on hemolysis for further colony processing. We cannot disregard the possibility that the discrepancy for the 48 specimens that were positive by the Aries GBS assay and negative by culture was due to nonhemolytic GBS colonies that were not detected by culture. Nonhemolytic GBS strains are only one of the challenges with culture-based screening, with a published sensitivity as low as 53% being the primary challenge (7). The overall labor cost for personnel performing cultures and a turnaround time slower than that of most NAATs are other deterrents for the use of culture for GBS screening. In theory, culture on chromogenic agars can produce results overnight after enrichment (11). However, in this study, 80% overgrowth on the SBAs plated after enrichment was observed and the samples had to be subcultured for isolation, adding another day of processing and more tech time to the work flow. Comparison of the Aries GBS assay results to the culture results yielded a sensitivity of over 95%, but the specificity (91.4%) and positive predictive value (72.1%) were affected by the use of enrichment culture as the reference standard. It is important to note that the use of culture as a reference standard introduces limitations for comparisons and assessment of sensitivity and specificity due to the subpar performance of the method itself (6). However, culture is still currently recognized as the gold standard for GBS surveillance under CDC guidelines (2). The use of NAATs as the gold standard by the FDA is not common yet. The introduction of more accurate and widely used NAATs for infectious diseases will likely lead to revision of the gold standard for *in vitro* diagnostic clinical trials in the future. Bidirectional sequencing showed agreement with the Aries GBS assay for 47 of the 53 specimens where the Aries GBS assay result was in disagreement with the reference culture method result. The sensitivity among other NAATs for GBS screening ranges from 95.4% for the Illumigene assay to 99.5% for the AmpliVue assay, with the sensitivities of the BD Max GBS assay and the Cepheid Xpert GBS LB assay being in between those values, placing the sensitivity of the Aries GBS assay equal to or better than that of other NAATs (19–22). Assessment of samples tested in parallel by the Aries GBS assay and either the BD Max GBS assay or the Cepheid Xpert GBS LB assay in this study determined a 100% correlation with the former method and a 98.0% correlation with the latter one.

This study focused on the use of the Aries GBS assay in pregnant women at 35 to 37 weeks of gestation; more studies are necessary to determine the performance of the assay on samples obtained during active premature labor. In conclusion, the Aries GBS assay is a sensitive, specific, and precise NAAT for GBS screening in preterm women. Its ease of use, short technician and turnaround times, and scalability make it a suitable option for use in either small community hospitals or large health care systems.
